# Tumor-Promoting Circuits That Regulate a Cancer-Related Chemokine Cluster: Dominance of Inflammatory Mediators Over Oncogenic Alterations

**DOI:** 10.3390/cancers4010055

**Published:** 2012-01-20

**Authors:** Tal Leibovich-Rivkin, Yosef Buganim, Hilla Solomon, Tsipi Meshel, Varda Rotter, Adit Ben-Baruch

**Affiliations:** 1 Department of Cell Research and Immunology, George S. Wise Faculty of Life Sciences, Tel Aviv University, Tel Aviv 69978, Israel; E-Mails: talinka1@gmail.com (T.L.-R.); tzipi@post.tau.ac.il (T.M.); 2 Department of Molecular Cell Biology, Weizmann Institute of Science, Rehovot 76100, Israel; E-Mails: yossi.buganim@gmail.com (Y.B.); hilla.besserglick@weizmann.ac.il (H.S.); varda.rotter@weizmann.ac.il (V.R.)

**Keywords:** Ras hyper-activation, p53dysfunction, cluster of cancer-related chemokines, inflammatory cytokines, TNFα, IL-1β

## Abstract

Here, we investigated the relative contribution of genetic/signaling components *versus* microenvironmental factors to the malignancy phenotype. In this system, we took advantage of non-transformed fibroblasts that carried defined oncogenic modifications in Ras and/or p53. These cells were exposed to microenvironmental pressures, and the expression of a cancer-related chemokine cluster was used as readout for the malignancy potential (CCL2, CCL5, CXCL8, CXCL10). In cells kept in-culture, synergism between Ras hyper-activation and p53 dysfunction was required to up-regulate the expression of the chemokine cluster. The *in vivo* passage of Ras^High^/p53^Low^-modified cells has led to tumor formation, accompanied by potentiation of chemokine release, implicating a powerful role for the tumor microenvironment in up-regulating the chemokine cluster. Indeed, we found that inflammatory mediators which are prevalent in tumor sites, such as TNFα and IL-1β, had a predominant impact on the release of the chemokines, which was substantially higher than that obtained by the oncogenic modifications alone, possibly acting through the transcription factors AP-1 and NF-κB. Together, our results propose that in the unbiased model system that we were using, inflammatory mediators of the tumor milieu have dominating roles over oncogenic modifications in dictating the expression of a pro-malignancy chemokine readout.

## 1. Introduction

The complex and multi-factorial nature of malignant tumors reflects the cooperative activities of intracellular transformation events and microenvironmental networks. While genetic and epigenetic alterations are essential for oncogenesis, it is now known that factors of the tumor milieu have a profound influence on the ability of transformed cells to establish tumors and to metastasize [1,2].

The cooperation between multiple elements in dictating tumor growth and progression makes it difficult to dissect the relative contribution of each of the partners to malignancy. Efforts that were made in this direction were hampered by the use of model systems based on transformed cells that carried “built in” intrinsic modifications in genetic and signaling pathways. On the background of the alterations already existing in these cells, it was difficult to determine the relative impact of isolated events on disease course.

In the present study, we have set an unbiased model system in which we have determined the relative contribution of oncogenic genetic/signaling components *versus* microenvironmental factors to the malignancy phenotype. Here, we have imposed defined oncogenic modifications on a normal cell system [3,4], and have applied microenvironmental constrains on these modified cells. Using this model system, we determined the relative effects of each of the two partners—oncogenic alterations *versus* microenvironmental factors—on the expression of chemokines that form a cancer-promoting network. The chemokines included in the network are characterized by being inflammatory chemokines that often act in parallel but through diverging mechanisms to promote malignancy phenotypes.

This network comprised of the chemokines CCL2, CCL5 and CXCL8 whose expression is predominantly up-regulated in many malignant diseases, and therefore their elevation manifests the acquisition of a more malignant phenotype by the cells. These three chemokines are classified as potent tumor-promoting chemokines in a very large number of malignancies, and their roles include, between others: Induction of high presence of Tumor-Associated Macrophages (TAM) in tumors (CCL2, CCL5); Elevation of angiogenesis (CCL2 and CXCL8); and induction of tumor cell migration and proliferation (CCL5, CXCL8) [5,6,7,8,9,10,11,12,13,14]. In parallel, we wished to know if similar ongcogenic/microenvironmental regulatory constrains will adhere to an inflammatory chemokine with more complex effects on malignancy, such as CXCL10. CXCL10 attracts Th1 and NK cells to tumor sites and inhibits angiogenesis, but in parallel can exert a variety of pro-cancerous functions [12,14,15]. For the sake of simplicity, in the following sections of the manuscript the four chemokines (CCL2, CCL5, CXCL8, CXCL10) will be referred together as “cancer-related chemokine cluster”.

To perform the above-mentioned analyses, we have used non-transformed fibroblasts carrying oncogenic modifications that are prevalent in many cancer diseases [3,4]: (1) Hyper-activation of the oncogenic Ras protein. It is now well-established that due to mutations in Ras or over-expression of receptor tyrosine kinases (RTKs), the Ras pathway becomes hyper-activated in tumor cells, leading to increased cell proliferation and survival [16,17,18]; (2) Down-regulation of the tumor-suppressing protein p53. Mutations in p53 or its allelic loss are frequently detected in malignancy, with deleterious effects ensued [19,20,21,22,23].

Using such modified fibroblasts which were kept in-culture, our study shows that both Ras hyper-activation and p53 down-regulation were required together in order to induce the expression of the cancer-related chemokine cluster; however, when such cells were exposed to the tumor microenvironment *in vivo*, the inflammatory milieu had a more powerful driving force towards a higher tumor-promoting phenotype than the genetic modifications, as manifested in this case by elevated levels of the cancer-related chemokine cluster. Specifically, we have identified Tumor Necrosis Factor α (TNFα) and Interleukin 1β (IL-1β)—two inflammatory mediators that are known to be of a tumor-promoting phenotype [24,25,26,27,28,29]—as potent chemokine inducers in the present cell system, and revealed that their effects were significantly higher than those obtained by the oncogenic alterations of Ras and p53 combined.

Taken together, by using an advantageous unbiased system based on non-transformed cells, our study shows that in this setup, the inflammatory microenvironment dominates over the effects of oncogenic alterations in aspects related to the expression of the cancer-related chemokine cluster, emphasizing the indispensible roles played by host factors in promoting malignancy-related events. These findings may have major clinical implications because they accentuate the need to consider the targeting of inflammatory mediators that are present at the tumor milieu when therapeutic measures are designed in malignant diseases.

## 2. Results and Discussion

### 2.1. Co-Expression of Hyper-Activated Ras and Dysfunctional p53 Together, in Non-Transformed Cells, Leads to Excessive Release of the Cancer-Related Chemokine Cluster

In this study, we have used WI-38 non-transformed fibroblasts as a platform to determine the effects of hyper-activated Ras and down-regulated p53 on the cluster of cancer-related chemokines. To this end, the cells were infected to express the constitutively active Ras^G12V^ mutant (Ras^High^ = Ras^H^) along with dysfunctional modalities in p53 that are prevalent in human malignant diseases: (1) p53 knock down by specific shRNA (p53^Low^ = p53^L^) that recapitulates the allelic loss of p53 or its abnormal degradation; (2) Over-expression of vectors expressing mutated p53, namely p53^R175H^ or p53^R248Q^, representing frequent tumor-promoting mutations of p53 in human cancers [19,20,21,22].

**Figure 1:**
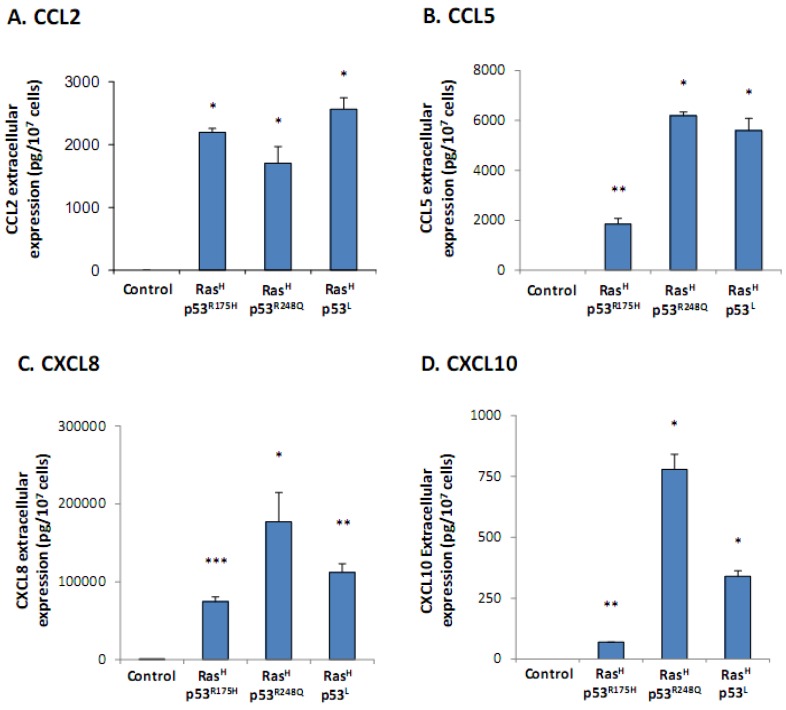
The release of the cancer-related chemokine cluster is up-regulated in non-transformed cells modified to express oncogenic Ras and dysfunctional p53. WI-38 human fibroblasts were modified to express oncogenic Ras and dysfunctional p53, together. These cells were kept in culture, and chemokine expression was determined in their supernatants by sandwich ELISA assays, at the linear range of absorbance. (**A**) CCL2. (**B**) CCL5. (**C**) CXCL8. (**D**) CXCL10. * *p* < 0.05, ** *p* < 0.01, *** *p* < 0.002 for differences between Ras/p53-modified cells and control cells, in which the expression of Ras and p53 was not modified. In all panels of the Figure, a representative experiment of at least n = 3 is presented.

The results of Figure 1 show that the combination of Ras hyper-activation + p53 dysfunction has induced a significant elevation in secretion of the members of the pro-malignancy chemokine network, consisting of CCL2, CCL5 and CXCL8, as well as of CXCL10 which is the chemokine showing more complex roles in malignancy (Figure 1A–D, respectively). Of the three modalities used for deregulation of p53, the p53^L^ (shRNA) and p53^R248Q^ forms were very effective in inducing the secretion of all chemokines, while the p53^R175H^ modality was much less efficient in most analyses and at times not active (see below). Overall, these results indicate that in cells that carry a normal phenotype, combined expression of the Ras and p53 oncogenic events shifts the balance in favor of a malignancy phenotype which is manifested by the elevated release of pro-cancerous chemokines.

### 2.2. The Release of the Cancer-Related Chemokine Cluster Requires Cooperation between Ras Hyper-Activation and p53 Down-Regulation

After determination of the effects induced by hyper-activated Ras and dysfunctional p53 on chemokine secretion, we wished to know if either oncogenic modification could act alone to increase the release of the chemokines, or whether cooperation between them is required. Because of its high effectiveness in promoting chemokine release when combined with Ras hyper-activation (Figure 1), in this part of the study we focused on the p53^L^ mode of p53 inactivation (by p53 shRNA).

**Figure 2:**
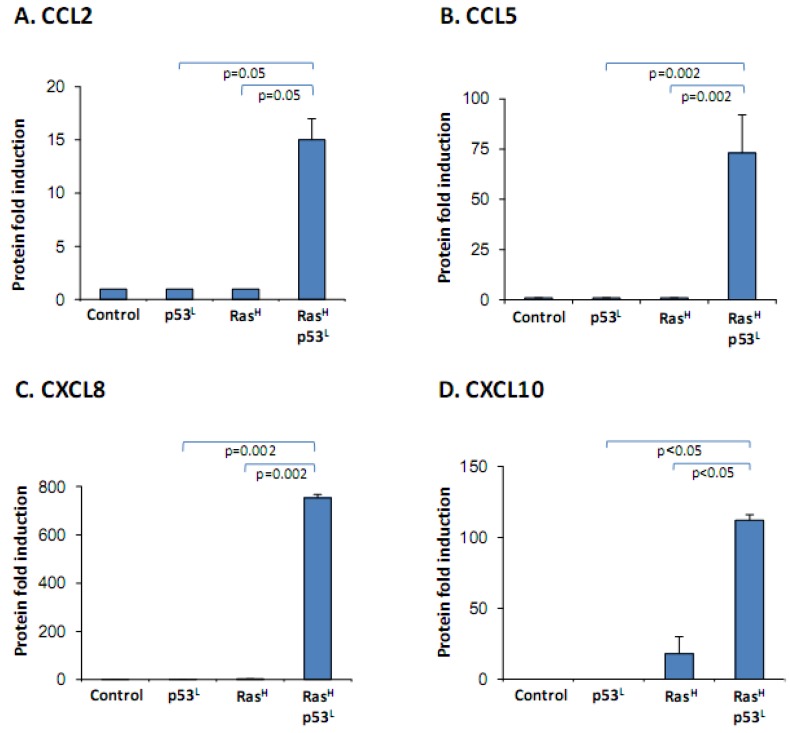
The release of the cancer-related chemokine cluster requires the synergistic activities of oncogenic Ras and dysfunctional p53. WI-38 human fibroblasts were modified to express oncogenic Ras and dysfunctional p53 together, or each alone. Chemokine expression was determined in supernatants of the different cell types by sandwich ELISA assays, at the linear range of absorbance. (**A**) CCL2. (**B**) CCL5. (**C**) CXCL8. (**D**) CXCL10. *p* values were obtained from the original OD values of these assays, prior to normalization. In all panels of the Figure, a representative experiment of at least n = 3 is presented.

To this end, we have expressed in the non-transformed cells the hyper-activated form of Ras and p53^L^, each alone or together. The results of Figure 2 show minimal effect of hyper-activated Ras alone (Ras^H^), or of knocked-down p53 alone (p53^L^) on chemokine secretion. However, when combined, Ras^H^ synergized with p53^L^, together leading to a prominent release of CCL2, CCL5, CXCL8 and CXCL10 by the cells.

These results indicate that cooperation between oncogenic modifications in Ras and p53 is required for induction of the cancer-related chemokine cluster. This finding shows that this chemokine-cluster readout obeys the same regulatory patterns observed for other tumor-promoting characteristics, in that its induction requires synergism between at least two oncogenic modifications [30,31,32,33,34,35,36,37,38]. The above findings also indicate that the chemokine cluster serves as an appropriate manifestation of the malignancy phenotype of the cells, and more specifically they point to the important roles played by hyper-activated Ras and dysfunctional p53 in regulation of the inflammatory setup of the tumor microenvironment, as indicated by the chemokine readout. Thus, acting together, hyper-activated Ras and dysfunctional p53 may give rise to exacerbated release of inflammatory chemokines with tumor-supporting functions, that can promote pro-cancerous activities: in tumor cells (originating due to genetic/signaling modifications), and in microenvironmental cells at their vicinity.

Also, our findings suggest that in patients exhibiting hyper-activation of the Ras pathway—due to Ras mutations and/or over-expression of RTKs—and also deregulated p53, chemokine release by the tumor cells would be promoted and the tumor microenvironment would be exposed to their tumor-supporting effects. This mode of regulation may be a part of a more complex and reciprocal net of interactions that exists between chemokines and oncogenic modifications, since recent findings have shown that over-expression of the chemokine receptor CXCR4—that is associated with tumor metastasis [39,40]—has led to up-regulation of RTKs and to deregulation of the p53-MDM2 axis [41].

### 2.3. Exposure to the Host Microenvironment Leads to Further Increase in the Expression of the Cancer-Related Chemokine Cluster

Published studies indicate that combination between oncogenic events can lead to complete transformation of cells, to the degree that they can establish full-blown tumors [30,31,32,33,34,35,36]. Along these lines, the Ras^H^ + p53^L^ cells that were used in our study, to be now termed “Ras^H^/p53^L^-in-culture” cells, were found to form tumors when inoculated to mice [3,4]. The gain of tumorigenic potential by these cells has motivated us to ask what would be the effect of exposure to host systems on the expression of the cancer-related chemokine cluster, in cells expressing oncogenic modifications.

**Figure 3:**
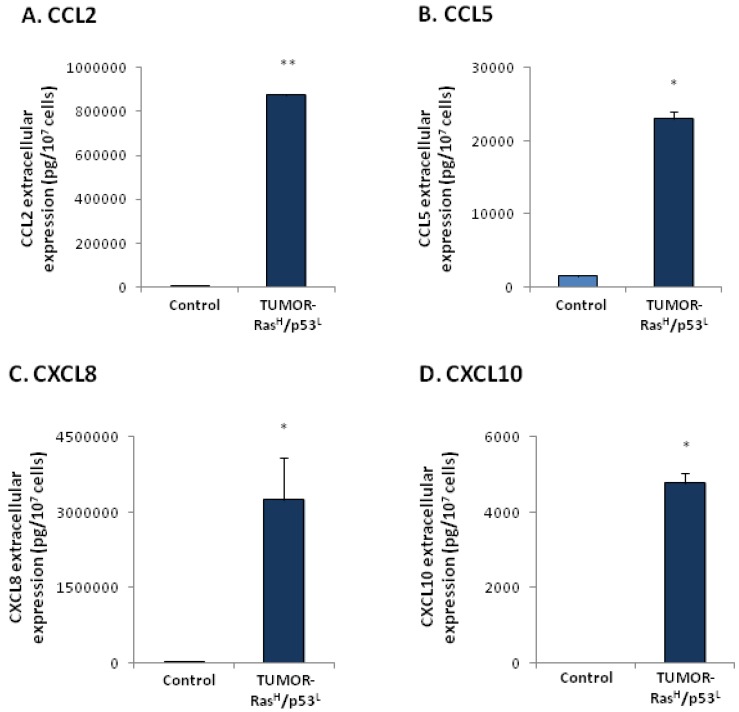
TUMOR-Ras^H^/p53^L^ cells release exacerbated levels of the cancer-related chemokine cluster. In-culture cells expressing oncogenic Ras and dysfunctional p53 (by shRNA), namely Ras^H^/p53^L^-in-culture cells, were inoculated to mice and formed tumors. Cells that have been excised from these tumors [4] were termed TUMOR-Ras^H^/p53^L^ cells. The expression of chemokines in supernatants of TUMOR-Ras^H^/p53^L^ cells was determined by sandwich ELISA assays, at the linear range of absorbance. (**A**) CCL2. (**B**) CCL5. (**C**) CXCL8. (**D**) CXCL10. * *p* < 0.05, ** *p* < 0.01 for differences between TUMOR-Ras^H^/p53^L^ cells and control cells, in which the expression of Ras and p53 was not modified. In all panels of the Figure, a representative experiment of at least n = 3 is presented.

**Figure 4:**
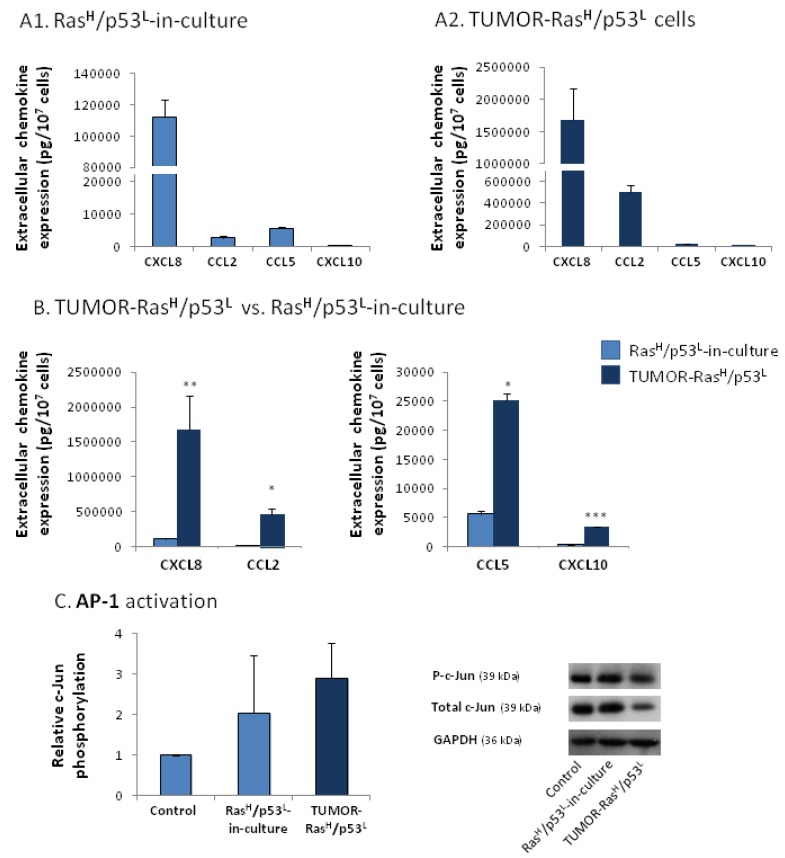
Exposure to the host microenvironment promotes the release of the cancer-related chemokine cluster, possibly through inflammation-related stimuli. (**A**, **B**) Chemokine expression was determined in supernatants of the cells by sandwich ELISA assays, at the linear range of absorbance. (**A1**) General relative pattern of chemokine release by Ras^H^/p53^L^-in-culture cells expressing oncogenic Ras and knocked-down p53. (**A2**) General relative pattern of chemokine release by TUMOR-Ras^H^/p53^L^ cells that were obtained by inoculation of Ras^H^/p53^L^-in-culture cells to mice. (**B**) Comparison between the levels of chemokines released by Ras^H^/p53^L^-in-culture cells and TUMOR-Ras^H^/p53^L^ cells. * *p* < 0.05, ** *p* < 0.01, *** *p* < 0.002 for differences between the two cell types. In parts (**A**) and (**B**), the results are representatives of at least n = 3. (**C**) C-Jun activation in TUMOR-Ras^H^/p53^L^ cells, in Ras^H^/p53^L^-in-culture cells and in control cells, in which the expression of Ras and p53 was not modified, determined by Western blot analysis. Relative c-Jun phosphorylation levels were calculated as described in the “Experimental Section”. The results are an average ± SD of n = 3.

To analyze this question, following the inoculation of the Ras^H^/p53^L^-in-culture cells to mice, cancer cells were excised from the tumors that have developed, and were brought back to culture [4]. The tumor cells, termed herein TUMOR-Ras^H^/p53^L^ cells, were found to exert a powerful elevation in the release of the cancer-related chemokines when compared to control non-modified cells (Figure 3). To assess the specific contribution of the tumor microenvironment to the expression of the chemokines, we have compared the TUMOR-Ras^H^/p53^L^ cells to their in-culture counterparts. This comparison revealed that exposure to the host microenvironment has further induced prominent elevation in the release of the chemokines (Figure 4A,B). In both cell types, CXCL8 was the most prominent chemokine released by the cells. Of note, the chemokine CXCL10 obeyed the regulatory pattern observed for the typical tumor-promoting chemokines CCL2, CCL5 and CXCL8, however in general it was released in lower levels than the other chemokines. The lower efficiency obtained for CXCL10 induction by the Ras and p53 modifications, as well as by the *in vivo* constrains, may reflect the more complex roles of this chemokine in cancer.

Taken together, these results indicate that malignant transformation due to co-expression of oncogenic modifications induces the expression of the cancer-related chemokines; however, the expression of the chemokines can be further promoted by exposure of the cells to elements residing at the tumor microenvironment. These findings emphasize the importance of host systems in selecting cells that have preferential expression of tumor-promoting traits, including gene products that affect angiogenesis and additional pro-cancerous activities, such as those attributed to the cancer-related chemokines [4,42,43,44,45].

### 2.4. The Exacerbated Release of the Chemokine Cluster Following Exposure to the Host Can be Recapitulated by Stimulation with Inflammatory Cytokines

The results shown in Figure 3 and Figure 4A–B actually show that TUMOR-Ras^H^/p53^L^ cells retained an amplified pattern of chemokine release when they were brought back to culture, indicating that they carry permanent modification/s which enable them, *in vitro*, to keep releasing exacerbated levels of the chemokines. Specifically, the high basal release of the cancer-related chemokine cluster by these cells may reflect the constitutive activation of transcription factors known to regulate such chemokines. To address this possibility, we have determined the activation level of AP-1, a key inducer of inflammatory chemokines in the context of immune activities [46,47,48], in TUMOR-Ras^H^/p53^L^ cells. Interestingly, we found that while the total levels of the c-Jun subunit of AP-1 has been consistently reduced in these cells, the phosphorylation level of the protein remained high, thus giving rise to elevated basal activation of AP-1 in these cells (Figure 4C). In comparison, no constant activation of c-Jun was detected in Ras^H^/p53^L^-in-culture cells (Figure 4C). Overall, the AP-1 transcription factor had a higher basal activation in the TUMOR-Ras^H^/p53^L^ cells that have emerged from the *in vivo* passage, than control cells in which the expression of Ras and p53 was not modified.

The elevated basal activation of AP-1 in TUMOR-Ras^H^/p53^L^ cells (Figure 4C) and the high chemokine amounts released by these cells (Figures 4A–B), point to the possibility that inflammatory mediators play key roles in regulating the inflammatory chemokines in Ras^H^/p53^L^ cells that were exposed to host systems *in vivo*. Inflammatory cytokines, such as Tumor Necrosis Factor α (TNFα) and Interleukin 1β (IL-1β) are known to elevate the release of inflammatory chemokines in the course of immune activities, and to activate the transcription factors AP-1 and NF-κB under such conditions [46,47,48,49,50,51,52,53,54,55,56]. Furthermore, these two cytokines were recently identified as tumor-promoting factors in many malignant diseases [24,25,26,27,28,29]. These published findings suggested that TNFα or IL-1β may be potential candidates whose activities *in vivo* may have led to the extremely high levels of the chemokine cluster in TUMOR-Ras^H^/p53^L^ cells.

At the tumor site, the sources for TNFα or IL-1β may be in host cells that are located in the tumor microenvironment, or in the tumor cells themselves. Therefore, we have determined the possibility that TUMOR-Ras^H^/p53^L^ cells have acquired the ability to permanently release TNFα and/or IL-1β, that then act in autocrine manners on the cells and induce the release of the inflammatory chemokines. Our data indicated that this was not the case, because TNFα or IL-1β were not secreted by the TUMOR-Ras^H^/p53^L^ cells (data not shown).

In view of the above, we raised an alternative possibility hypothesizing that the elevated levels of activated AP-1 in TUMOR-Ras^H^/p53^L^ cells actually attest for repeated selective pressures that were applied by an inflammatory tumor microenvironment in the host, leading eventually to selection of cells that carry high basal activation of transcription factor/s that promote the expression of the chemokines. In such a case, it is possible that the TUMOR-Ras^H^/p53^L^ cells manifest high responsiveness to inflammatory cytokines, which has led to the activation of AP-1.

**Figure 5:**
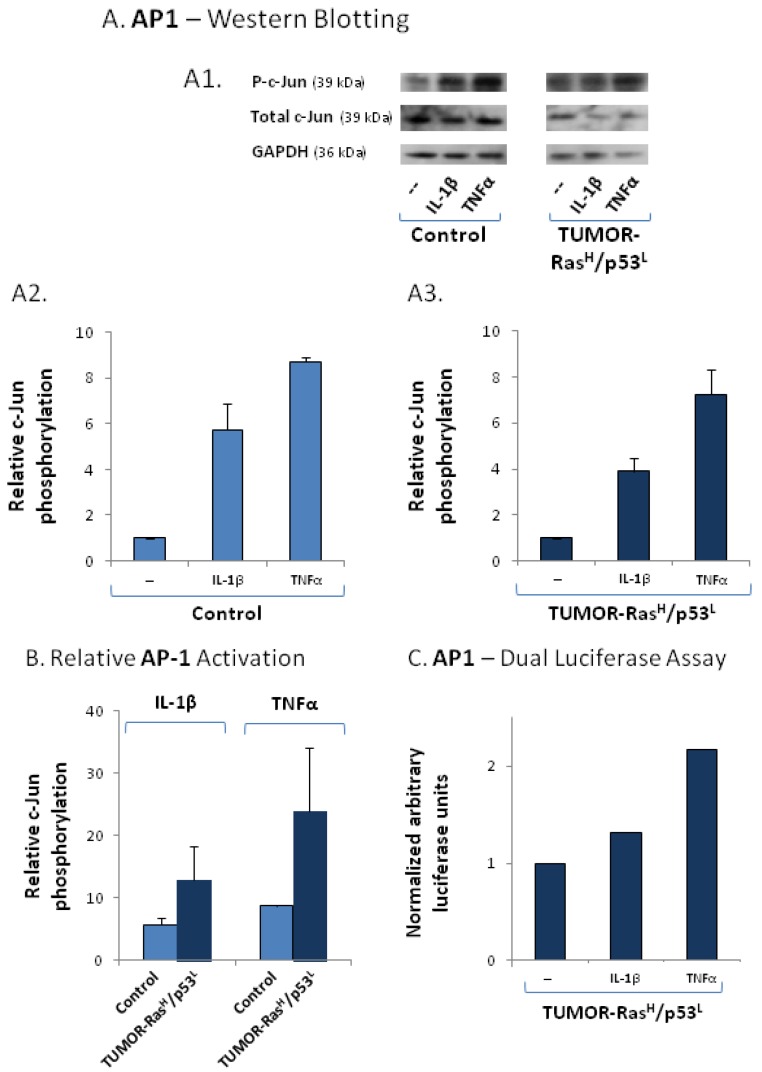
TUMOR-Ras^H^/p53^L^ cells are highly responsive to inflammatory stimulation, as indicated by AP-1 activation. (**A**) AP-1 activation was determined by analyzing c-Jun phosphorylation levels in TUMOR-Ras^H^/p53^L^ cells and in control cells (in which the expression of Ras and p53 was not modified), prior and following stimulation by IL-1β (500 pg/mL) or TNFα (50 ng/mL) for 24 h, by Western blot analysis. (**A1**) Western blots showing c-Jun phosphorylation, total c-Jun levels and GAPDH levels as a loading control. A representative experiment of n = 2 is presented. (**A2**) Densitometry values of control cells, prior or after stimulation by the cytokines. Non-stimulated cells were given the value of 1. (**A3**) Densitometry values of TUMOR-Ras^H^/p53^L^ cells, prior or after stimulation by the cytokines. Non-stimulated cells were given the value of 1. In (**A2**) and (**A3**), relative AP-1 activation levels were calculated as described in the “Experimental Section” (phosphorylated c-Jun/total c-Jun/GAPDH) and the results presented are the average ± SD of densitometry levels obtained in 2 experiments. (**B**) Relative levels of c-Jun phosphorylation induced by IL-1β or TNFα in control cells, in comparison to TUMOR-Ras^H^/p53^L^ cells. The values were obtained by comparison of the densitometry values of cells (control or TUMOR-Ras^H^/p53^L^) treated by the cytokines, to non-stimulated control cells, that were given the value of 1 (the control cells are not shown in the Figure). The results are the average ± SD of activation levels obtained in 2 experiments. (**C**) TUMOR-Ras^H^/p53^L^ cells were transfected with (1) construct of firefly luciferase under the control of AP-1 binding sites, and (2) construct of renilla luciferase. The cells were either not stimulated or stimulated with IL-1β (500 pg/mL, 6–8 h) or TNFα (50 ng/mL, 8 h). Time points were selected based on preliminary kinetics analysis. Luciferase AP-1 activation levels relative to renilla luciferase in non-stimulated cells were given the value 1. A representative experiment of n = 3 is presented.

Supporting this hypothesis were the findings of Figure 5, showing analyses of TUMOR-Ras^H^/p53^L^ cells following stimulation by TNFα or IL-1β. Measuring the activation of the AP-1 subunit c-Jun, we found that IL-1β and more potently TNFα activated the AP-1 signaling pathway in TUMOR-Ras^H^/p53^L^ cells and in control cells (Figure 5A). However, the results of Figure 5B show that the overall phosphorylation levels of c-Jun in TUMOR-Ras^H^/p53^L^ cells was higher than in control cells. Because we could not obtain sufficient reduction of c-Jun expression by siRNA (data not shown), we have determined the direct ability of IL-1β and TNFα to activate AP-1 by using a dual luciferase assay with AP-1 reporter. These assays were performed only on the TUMOR-Ras^H^/p53^L^ cells, because they were showing higher responsiveness to the cytokines than control cells, and have indicated that the two cytokines, and primarily TNFα induced the activation of this pathway in the TUMOR-Ras^H^/p53^L^ cells )Figure 5C).

**Figure 6:**
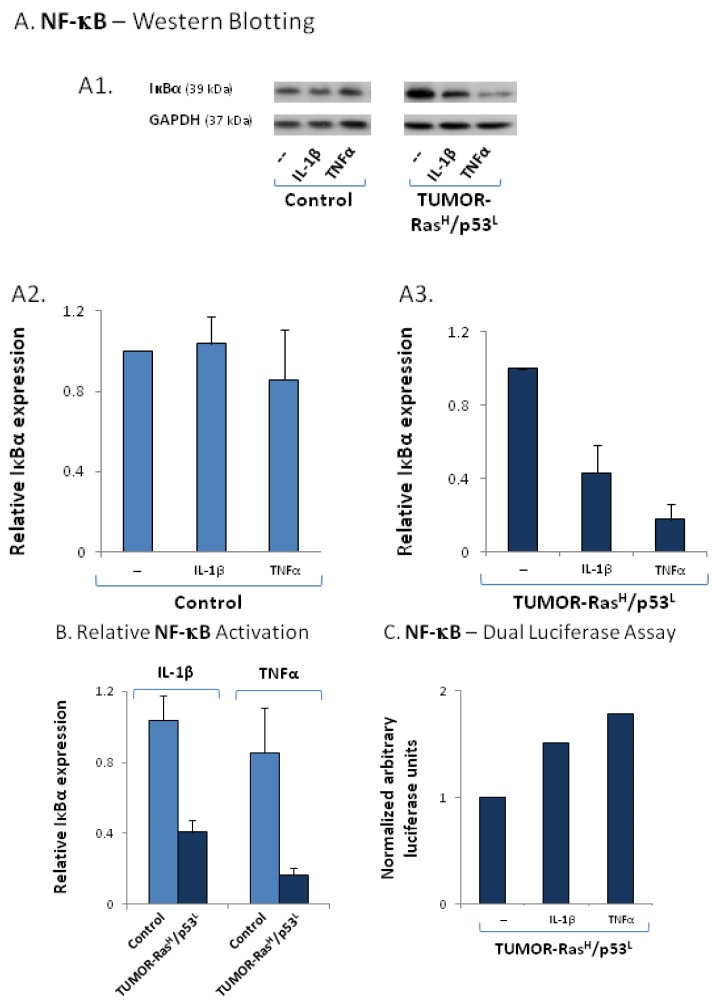
TUMOR-Ras^H^/p53^L^ cells are highly responsive to inflammatory stimulation, as indicated by NF-κB activation. (**A**) NF-κB activation was determined by analyzing IκBαlevels in TUMOR-Ras^H^/p53^L^ cells and in control cells (in which the expression of Ras and p53 was not modified), prior and following stimulation by IL-1β (500 pg/mL) or TNFα (50 ng/mL) for 24 h, by Western blot analysis. (**A1**) Western blots showing IκBα levels, and GAPDH levels as a loading control. A representative experiment of n = 3 is presented. (**A2**) Densitometry values of control cells, prior or after stimulation by the cytokines. Non-stimulated cells were given the value of 1. (**A3**) Densitometry values of TUMOR-Ras^H^/p53^L^ cells, prior or after stimulation by the cytokines. Non-stimulated cells were given the value of 1. In (**A2**) and (**A3**), relative IκBα levels were calculated (IκBα levels/GAPDH), and the results present the average ± SD of densitometry levels obtained in 3 experiments. (**B**) Relative levels of IκBα induced by IL-1β or TNFα in control cells, in comparison to TUMOR-Ras^H^/p53^L^ cells. The values were obtained by comparison of the densitometry values of cells (control or TUMOR-Ras^H^/p53^L^) treated by the cytokines, to non-stimulated control cells, that were given the value of 1 (the control cells are not shown in the Figure). The results are the average ± SD of activation levels obtained in 3 experiments. (**C**) TUMOR-Ras^H^/p53^L^ cells were transfected with (1) construct of firefly luciferase under the control of NF-κB binding sites, and (2) construct of renilla luciferase. The cells were either not stimulated or stimulated with IL-1β (500 pg/mL, 6–8 h) or TNFα (50 ng/mL, 8 h). Time points were selected based on preliminary kinetics analyses. Luciferase NF-κB activation levels relative to renilla luciferase in non-stimulated cells were given the value 1. A representative experiment of n = 3 is presented.

In parallel, we have determined the activation of the NF-κB pathway, first asking if TNFα and IL-1β reduce the levels of the IκBα inhibitor of NF-κB in control and TUMOR-Ras^H^/p53^L^ cells. Using the IκBα readout, we found that TNFα and IL-1β acted primarily on the TUMOR-Ras^H^/p53^L^ cells to reduce IκBα levels, with TNFα showing more prominent effects compared to IL-1β (Figure 6A). The results of Figure 6B show that the effects of the cytokines on IκBα levels were more prominent in TUMOR-Ras^H^/p53^L^ cells than in control cells. These results suggest that the two cytokines activate NF-κB in TUMOR-Ras^H^/p53^L^ cells. Support for this possibility was provided by dual luciferase assays with NF-κB reporter, showing the activation of this pathway by both cytokines, with higher activation achieved by the TNFα stimulation (Figure 6C).

Overall, the TUMOR-Ras^H^/p53^L^ cells, which were exposed to host-microenvironmental factors *in vivo* have shown preferential response to TNFα or IL-1β stimulation, possibly by activating both NF-κB and AP-1 signaling pathways.

The above results suggest that the TUMOR-Ras^H^/p53^L^ cells attest for processes that may take place *in vivo*, in which the activity of the inflammatory cytokines induces in the cells a specific setup and transcriptional up-regulation that eventually lead to elevated release of the inflammatory chemokines at the tumor site. To recapitulate this *in vivo* process, we exposed the cells that were modified to express oncogenic Ras and p53 and were kept in-culture, to the inflammatory mediators TNFα and IL-1β. Here, of the four chemokines we chose to focus on CCL2 and CCL5 because together their effects represent a broad spectrum of cancer-related activities that are exerted by the chemokines included in the cluster studied in this research.

**Figure 7:**
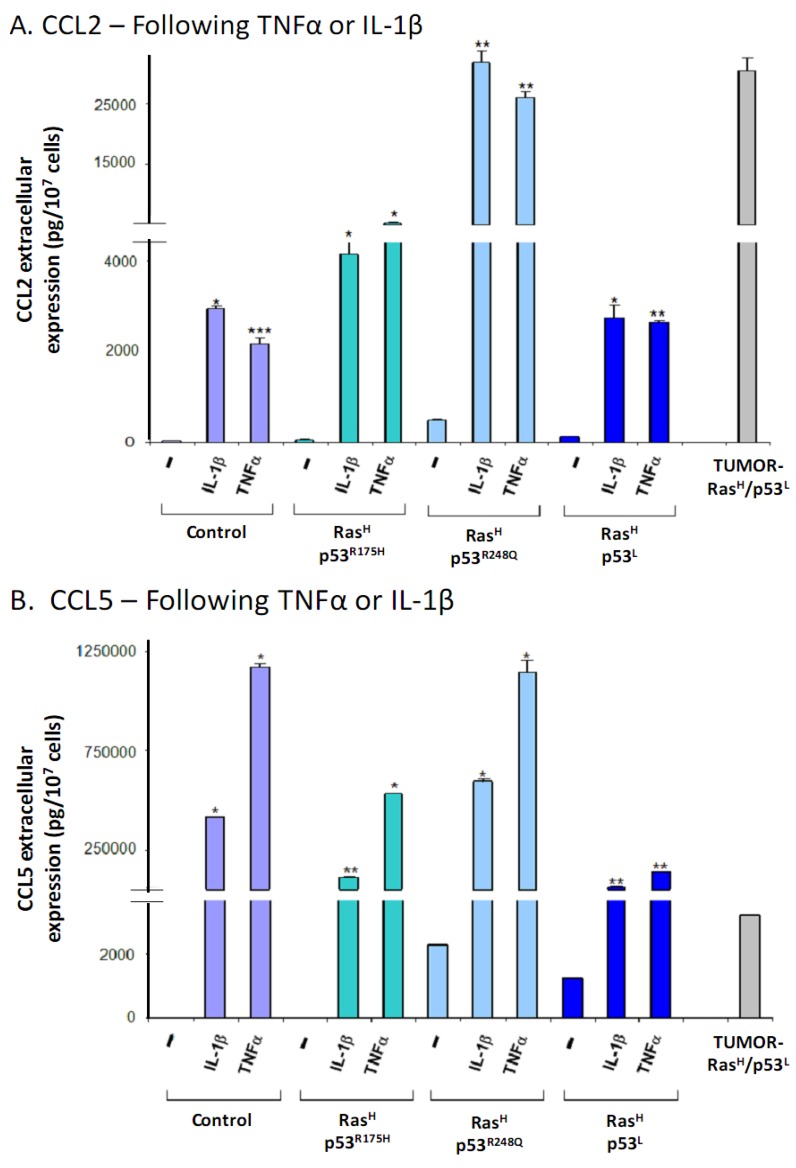
Inflammatory cytokines potentiate the release of cancer-related chemokines by cells expressing Ras^H ^and down-regulated p53 and kept in-culture, to the extent observed in TUMOR-Ras^H^/p53^L^ cells. Cells carrying both Ras-hyper-activation and p53 deregulation were stimulated by IL-1β (500 pg/mL) or TNFα (50 ng/mL) for 24 h, or not-stimulated. Chemokine expression was determined in supernatants of these cells, of control non-modified cells and of TUMOR-Ras^H^/p53^L^ cells by sandwich ELISA assays, used at the linear range of absorbance. (**A**) CCL2. (**B**) CCL5. * *p* < 0.05, ** *p* < 0.01 for differences between cytokine-stimulated cells and non-stimulated cells. A representative experiment of n = 3 is presented (except for non-modified control cells in which n = 2).

Specifically, we used cells that expressed Ras^H^ together with the different modifications of p53: p53^R175H^, p53^R248Q^ or p53^L^. In all of these cells, the stimulation by the inflammatory cytokines has led to increased CCL2 and CCL5 secretion which was way beyond the one obtained by the combined oncogenic modifications of hyper-activated Ras and deregulated p53 together (Figure 7A,B, respectively). Moreover, the effect of the cytokines was obtained regardless of the p53 modification used, because potent induction of the chemokines by TNFα and IL-1β was obtained in all forms of deregulated p53, namely p53^R175H^, p53^R248Q^ and p53^L^. Of note, the chemokine amounts obtained by stimulation of the different cell types kept in culture, expressing the oncogenic modifications, with the cytokines, were in general similar to those of TUMOR-Ras^H^/p53^L^ cells, or even higher (depending on the chemokine type).

An important point that was revealed by these analyses is that the inflammatory cytokines induced the release of the cancer-related chemokine cluster to greater extent than the combination of deregulated Ras and p53 together, and acted powerfully also without the oncogenic modification, as indicated by their ability to potently induce the release of CCL2 and CCL5 by control cells that were not modified with Ras hyper-activation and p53 down-regulation. Based on all the above findings, our conclusion is that when the chemokine cluster is used as readout for a malignancy phenotype, the tumor microenvironment has a primary role in dictating the malignancy trait of the cells. Nevertheless, when *in vivo* settings are concerned, it is possible that interactions between the inflammatory milieu and the oncogenic modifications would lead to a vicious cycle that potentiates the release the cancer-related chemokines.

## 3. Experimental Section

### 3.1. Cells

In this study, WI-38 human lung fibroblasts were used, following immortalization by hTERT as previously described [3,4]. These cells originally express wild type (WT) Ras and WT p53, and they were infected with the following constructs, as previously described [3,4]: Ras^High^ = Ras^H^—A constitutively active human H-Ras^G12V^ mutant expressed by a hygromycin-expressing vector; p53 shRNA (p53^Low^ = p53^L^), p53^R175H^, p53^R248Q^ expressed by puromycin-expressing vectors. Controls included WI-38 cells infected with the empty vector of hygromycin and with a puromycin-expressing vector (shRNA against the mouse but not the human NOXA gene was used as control for shRNA of p53).

In parallel to the cell lines kept in culture, cells expressing H-Ras^G12V^ and p53^L^ (termed “Ras^H^/p53^L^-in-culture”) were inoculated to nude mice [4]. The cells gave 100% incidence of local tumor uptake (without metastases), and cells obtained from such a tumor have been brought back to culture [4]. In contrast to primary murine host cells that cannot survive the continuous growth in culture (such as macrophages or fibroblasts), the cells excised from the tumor have given rise to a cell line that could be continuously grown *in vitro*, termed herein “TUMOR-Ras^H^/p53^L^”. Pathologic examination determined the sarcoma type of the resulting cells [4], having high mitotic rate.

**Supplementary Figure 1:**
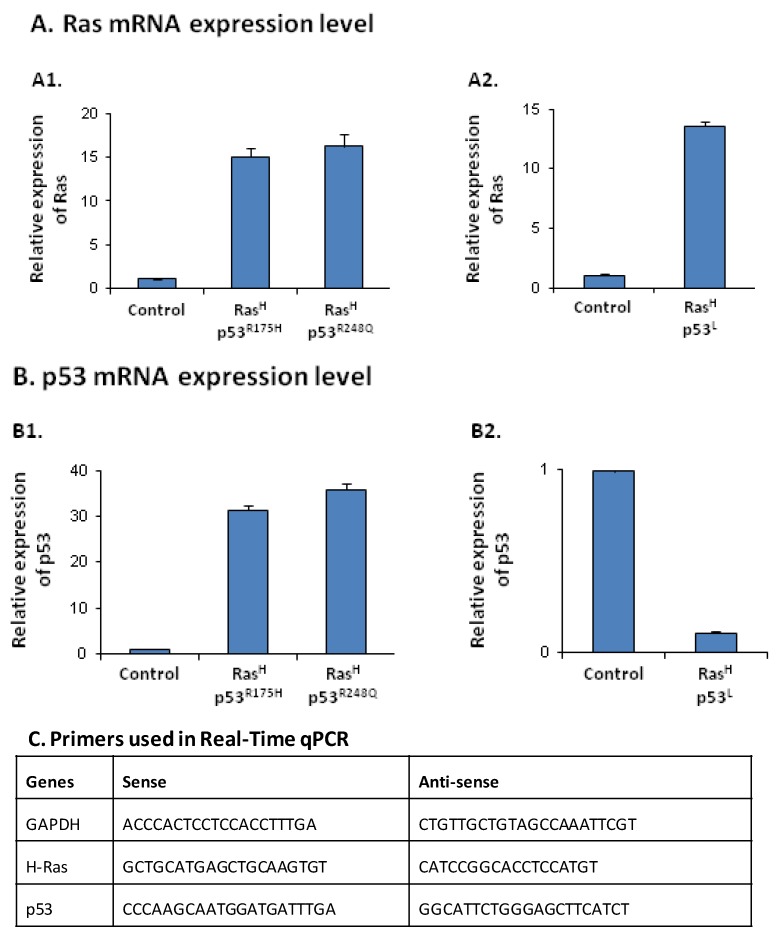
The expression levels of Ras and p53 mRNAs were determined in the different cell lines by qPCR. (**A**) Ras expression levels, normalized relatively to the levels obtained in cells expressing control vectors. (**A1**) Elevated levels of Ras in cells expressing Ras^H^/p53^R175H^ and Ras^H^/p53^R248Q^. (**A2**) Elevated levels of Ras expression in cells expressing Ras^H^/p53^L^ (Ras^H^/p53^L^-in-culture). (**B**) p53 expression levels, normalized relatively to the levels obtained in cells expressing control vectors. (**B1**) Elevated levels of p53 in cells over-expressing p53^R175H^ (Ras^H^/p53^R175H^) or p53^R248Q^ (Ras^H^/p53^R248Q^).(**B2**) Reduced levels of p53 in cells expressing shRNA to p53 (Ras^H^/p53^L^-in-culture). (**C**) The sequences of primers used for qPCR. These primers detect sequences shared by the WT and mutants. In panels (**A**) and (**B**), a representative experiment of at least n = 3 is presented.

Up-regulation of Ras expression and down-regulation of p53 expression were verified in all cell lines by real time qPCR (Supplementary Figure 1; Verification for some of the cell lines by Western blotting was also provided in [4]). All cell lines, including TUMOR-Ras^H^/p53^L ^cells, had similar growth rates in culture (data not shown).

### 3.2. Determination of Chemokine Release by ELISA

The different cell types, as appropriate, were cultured in growth medium. Then, the cells were washed twice in PBS and incubated overnight in Low Protein Medium (LPM) containing TNFα (50 ng/mL; 300-01A) or IL-1β (500 pg/mL; 200-01B) (PeproTech, Rocky Hill, NJ, USA). Cytokine concentrations were selected based on titration analyses performed in other studies in the laboratory. Control cells were grown under similar conditions, with 0.1% BSA, the solubilizer of TNFα and IL-1β.

Chemokine expression in the supernatants of the cells was determined by sandwich ELISA, at the linear range of absorbance, using recombinant human proteins for generation of standard curves. As expected of a biological system, there were fluctuations in the levels of chemokines released by the same cell line in the various assays. However, it is important to note that the general ratios between the secretion levels of the different cell types used in this study, as well as the ratios between the different chemokines in the same cell line, were kept proportionate in the different assays along the whole study.

The following antibodies were used: Coating antibodies—mouse monoclonal antibodies against human CCL2 (500-M71, PeproTech), CCL5 (500-M75, PeproTech), CXCL8 (508402; BioLegend, San Diego, CA, USA) and CXCL10 (500-P93, PeproTech); Detecting antibodies—biotinylated goat antibodies against human CCL2 (500-P34Bt, PeproTech), CCL5 (BAF278, R&D Systems), CXCL8 (BAF208; R&D Systems, Minneapolis, MN, USA) and CXCL10 (500-P93Bt, PeproTech). After the addition of streptavidin-horseradish peroxidase (HRP, Jackson ImmunoResearch Laboratories, West Grove, PA, USA), the substrate TMB/E solution (Chemicon, Temecula, CA, USA) was added. The reaction was stopped by the addition of 0.18 M H2SO4 and optical density (OD) was measured at 450 nm. *p* values were calculated by Student’s *t* test.

### 3.3. Western Blot Analyses

The different cell types, as appropriate, were lysed in RIPA lysis buffer. Cell lysis was followed by conventional Western blot procedures. The following proteins were analyzed by the appropriate antibodies: phosphorylated c-Jun (sc-822; Santa Cruz Biotechnology, Santa Cruz, CAF), c-Jun (610326; BD Transduction Laboratories, San Jose, CA), IκBα (4814; Cell Signaling Technology) and GAPDH (MAB374; Chemicon).

After washings, the membranes were incubated with horseradish peroxidase (HRP)-conjugated secondary antibodies, as appropriate: sheep anti-mouse-HRP (NA931; Amersham Pharmacia Biotech, Buckinghamshire, UK) or goat anti-rabbit-HRP (111-035-003; Jackson ImmunoResearch Laboratories). The membranes were subjected to enhanced chemilluminescence (Amersham), and bands on immunoblots were quantified by densitometry. Because of consistent reduction in c-Jun expression levels (total c-Jun) in TUMOR-Ras^H^/p53^L^ cells, “Relative c-Jun phosphorylation levels” were calculated by dividing the densitometry values obtained for phosphorylated-c-Jun, by the values of total c-Jun, and then by GAPDH which served as a loading control (phosphorylated c-Jun/total Jun/GAPDH).

### 3.4. Dual Luciferase Assays

The cells were transfected with vectors containing a luciferase gene under the control of an AP-1 binding sites (5 × coll-TRE-tata; kindly provided by Tsaffrir Zor, Tel Aviv University), or NF-κB binding sites (3 × KBL; kindly provided by Stefan Wiemann, DKFZ Heidelberg, Germany). A construct coding for renilla luciferase was used for normalization of the results according to transfection yields (kindly provided by Tsaffrir Zor, Tel Aviv University). 48 h after transfection, the cells were stimulated by TNFα (8 h) or IL-1β (6–8 h) (time points were selected following a kinetic analysis), and processed with the reagents provided in Dual-Luciferase Assay System Kit (Promega, Madison, WI, USA). Luciferase activity was determined using the same kit, according to manufacturer’s instructions.

## 4. Conclusions

This study has analyzed the contribution of oncogenic events as compared to inflammatory mediators to acquisition of a pro-tumorigenic phenotype, using a cancer-related chemokine cluster as readout for the malignancy potential of the cells. We have identified two independent steps in which the chemokine content may be regulated. At the first stage, combined oncogenic modifications lead to full transformation of the cells and is accompanied by increased release of the chemokine cluster. The second stage takes place when the tumor cells start interacting with their surroundings *in vivo*, and are affected by the tumor microenvironment. At this latter stage, inflammatory cytokines that are found at the scene have major impact on the release of the chemokines by the tumor cells, and this is manifested by exacerbated levels of pro-malignancy chemokines that are released by the transformed cells. Based on published findings, it is possible that the inflammatory cytokines are released by inflammatory cells or stroma cells that reside at the tumor site. In parallel, it is possible that in some of the cancer types, the tumor cells may constitute another source for the inflammatory cytokines, as was shown to be the case for example in breast cancer [24,25,26,27,28,29,57]. Of note is the fact that in many systems, tumor cells that are grown *in vitro* lose the ability to constitutively release the cytokines, as may have been the case for the TUMOR-Ras^H^/p53^L^ cells used in our study.

Importantly, this study delineates the relative contribution of each of the two steps to regulation of the cancer-related chemokine cluster and thus potentially to malignancy, by using a platform based on cells that are non-transformed, allowing us to clearly decipher the roles of each arm to the readout used. This study provides novel findings showing, by the use of this advantageous system, that the inflammatory microenvironment has a very strong impact on the expression levels of the cancer-related chemokines, that in the unique system we used, is higher than the ability of the oncogenic alterations to increase the expression of the pro-malignancy chemokines. Nevertheless, the roles of the genetic/signaling modifications is indispensable, because *in vivo* they are expected to enhance the vicious cycle taking place between cancer cells and host cells at their microenvironment.

Based on the above, we suggest that in the setup of our current study, the powerful chemokine-promoting activities of inflammatory cytokines dominate the regulatory events that may control chemokine release, and dictates the expression of the chemokines at the tumor microenvironment. These chemokines, in turn, lead to a large array of tumor-promoting activities, e.g., high presence of TAM in the tumors, angiogenesis, tumor cell invasion and more. In such a setting, inflammatory cells that were recruited to the tumor site in response to chemokines such as CCL2 and CCL5 continue releasing the inflammatory cytokines TNFα and IL-1β, whose activities further promote the release of the cancer-related chemokines. By that, a vicious cycle of pro-malignancy activities ensues, supporting processes of tumor growth and progression.

These findings may have important clinical implications because they provide major support to the significant power of the inflammatory tumor microenvironment and its being a potential target for therapeutic approaches. They also emphasize the importance of current efforts to introduce to the treatment of malignant diseases novel treatments, in which inflammatory mediators are targeted, for example by direct inhibition of TNFα activities. To date, measures that target TNFα activities are already used in the clinic, primarily in inflammatory diseases such as rheumatoid arthritis, motivating researchers to determine their impact on malignant diseases. So far, therapies directed against TNFα have been implemented in a limited number of malignant diseases [58,59,60], with proven safety. At this point, it is difficult to assess the actual effects of these treatments on disease course, because the studies were preliminary and were performed on patients with advanced/metastatic diseases, including such that have failed to respond to conventional therapies. However, our findings and other studies showing the great impact of inflammatory mediators on malignancies, strengthen the need to provide improved understanding of the joint activities of inflammatory mediators at tumor sites, and to evaluate the ability to use therapies against such factors in cancer.
